# Use of ROV for assessing marine litter on the seafloor of Saronikos Gulf (Greece): a way to fill data gaps and deliver environmental education

**DOI:** 10.1186/s40064-015-1248-4

**Published:** 2015-08-28

**Authors:** C. Ioakeimidis, G. Papatheodorou, G. Fermeli, N. Streftaris, E. Papathanassiou

**Affiliations:** Hellenic Centre for Marine Research, Institute of Oceanography, 46.7 km Athens-Sounio Ave., 19013 Anavyssos, Greece; Laboratory of Marine Geology and Physical Oceanography, Department of Geology, Faculty of Science, University of Patras, 26500 Patras, Greece; Institute of Educational Policy, Ministry of Culture, Education and Religious Affairs, Athens, Greece

**Keywords:** Litter, ROV, Plastic, Greece, Children, Education

## Abstract

A visual census of marine litter on the seafloor of the Saronikos Gulf (Greece) is presented in the current work. The abundance and qualitative composition of benthic marine litter were investigated in two selected locations of the Saronikos Gulf with a Remote Operated Vehicle, where other sampling strategies couldn’t be implemented. The assessment of marine litter was combined with environmental education within the PERSEUS (FP7) Research Project, in a novel 2-day research cruise where schoolchildren actively participated. Two transects of total length 2.36 km were carried out. A relevant marine litter protocol was developed where marine litter was categorized into six different categories according to their material type and where possible, the source of the items was identified. Plastics (55 %) and metals (36 %) had the biggest share among the recorded marine litter items. Marine litter proved to be an ideal theme in order to enhance the environmental awareness of schoolchildren.

## Background

Marine litter is one of the major environmental threats of the twenty-first century (UNEP 2011). The growing amounts of generated litter and the slow degradation rate result in the accumulation of litter in the oceans, posing a serious threat for the healthy oceans; indeed litter has been found widespread in the marine environment (Barnes et al. 2009). Benthic marine litter tends to become trapped in areas of low water circulation, high sediment accumulation and convenient seafloor morphology in contrast to floating litter, which accumulates in frontal areas (Galgani et al. 2000; Eriksen et al. 2014). Litter that reaches the seafloor may already have been transported over considerable distances, only sinking when weighed down by entanglement and fouling (Carpenter et al. 1972; Barnes and Milner 2005; Goldstein et al. 2012). The result is the accumulation of litter on specific seafloor locations depending on local sources and oceanographic conditions, which further lead to high spatial variability of marine litter abundance (Ramirez-Lolodra et al. 2013; Pham et al. 2013).

The most common approach to evaluate seafloor litter distribution in the shallow water (0–25 m), is to conduct underwater visual surveys with SCUBA diving (Katsanevakis and Katsarou 2004). Lundqvist (2013) proposed and used a towed video camera for marine litter surveys in shallow waters when diving was unsuitable, difficult or impossible due to inadequate circumstances (e.g. heavy marine traffic, cold water temperature). In the marine shelf environment, the most commonly used technique for surveying and assessing benthic litter is the opportunistic sampling using otter trawls, with several studies using this method (Stefatos et al. 1999, Galgani et al. 2000; Koutsodendris et al. 2008; Ioakeimidis et al. 2014). In the last years, the use of observation tools (Remote Operated Vehicles, ROV) in the deep-sea environment has also been introduced; despite the need for considerable resources (Schlining et al. 2013; Debrot et al. 2014; Vieira et al. 2014; Pham et al. 2014).

Much of the existing data on benthic marine litter comes from trawl surveys conducted by commercial trawl fisheries while the data derived from visual means are focused mostly on deep water. Therefore, benthic marine litter surveys, most of the times, have been limited to the commonly used fishing grounds and little is known about the marine litter abundance not only in deep-sea but also in shallow water, deeper than the limit of scuba diving and beyond the fishing grounds. In shallow waters such data (Scuba and ROVs) are sparse and limited.

This is the case of the Saronikos Gulf where Ioakeimidis et al. (2014) monitored benthic marine litter by using commercial trawl fisheries for a significant part of the gulf (50–350 m water depth) and Katsanevakis and Katsarou (2004) recorded benthic marine litter in shallow water (<25 m) by using scuba diving. Nonetheless, marine litter could not be assessed for areas of Saronikos Gulfs where commercial trawl fisheries are excluded and thus other means and methodologies should be implemented.

Albeit the fact that nowadays there is excess of information related to the problem of marine litter and its side effects onto the environment, people are still littering. The alteration of the consumer behavior might be a key point towards containing the problem of marine litter. Towards that direction, enhancing environmental awareness in children is always a challenge. Children do worry about the environment and are highly motivated when are convinced that they could effectively protect it and be part of the change (Schreiner et al. 2005; Evans et al. 2007). Among the various environmental thematics, marine litter poses a problem for which children are highly concerned. Hartley et al. (2015) used marine litter in a dedicated environmental educational project, which was carried out by nine high schools in SW England.

The present study aims to quantify the amount of litter being present on the seafloor of a semi-enclosed gulf by the use of ROV. The study locations were selected in shallow waters, much deeper than the scuba diving limit and beyond the fishing grounds. The ultimate scope is to get a better view of the marine litter distribution on the seafloor of the Saronikos Gulf, while also taking into account existing studies. Up to our knowledge, no relevant data exist deriving from ROV surveys for the Eastern Mediterranean. This study also introduces another dimension in which marine research could work synergistically in with environmental education.

## Setting-up an educational marine research cruise

### Participation in “Andromeda I”

A two-day novel educational marine research cruise was organized under the code name “Andromeda I”, in conjunction with the PERSEUS (FP7) Research Project’s educational network “My school voyages with PERSEUS”. Among 600 schoolchildren, which systematically work under the environmental education network “My School voyages with PERSEUS”, a selection of 20 schoolchildren and 10 teachers was done. In total, 10 different schools from Greece and Italy participated in the educational research cruise.

During the “Andromeda I” educational cruise, schoolchildren studied basic aspects of the marine environment, among which was the recording of marine litter on the seafloor of the Saronikos Gulf with the use of robotics.

Schoolchildren were invited to answer comprehensive and age-appropriate questions such as:

- Why is marine litter a problem for the seas? (Children awareness)
- How bad is marine litter for the seas? (Perceived impacts)
- What are the main sources of marine litter? (Perceived causes)
- Is plastic litter the most abundant type of litter in the seas? (Plastic litter)

### Survey design

**Fig. 1:**
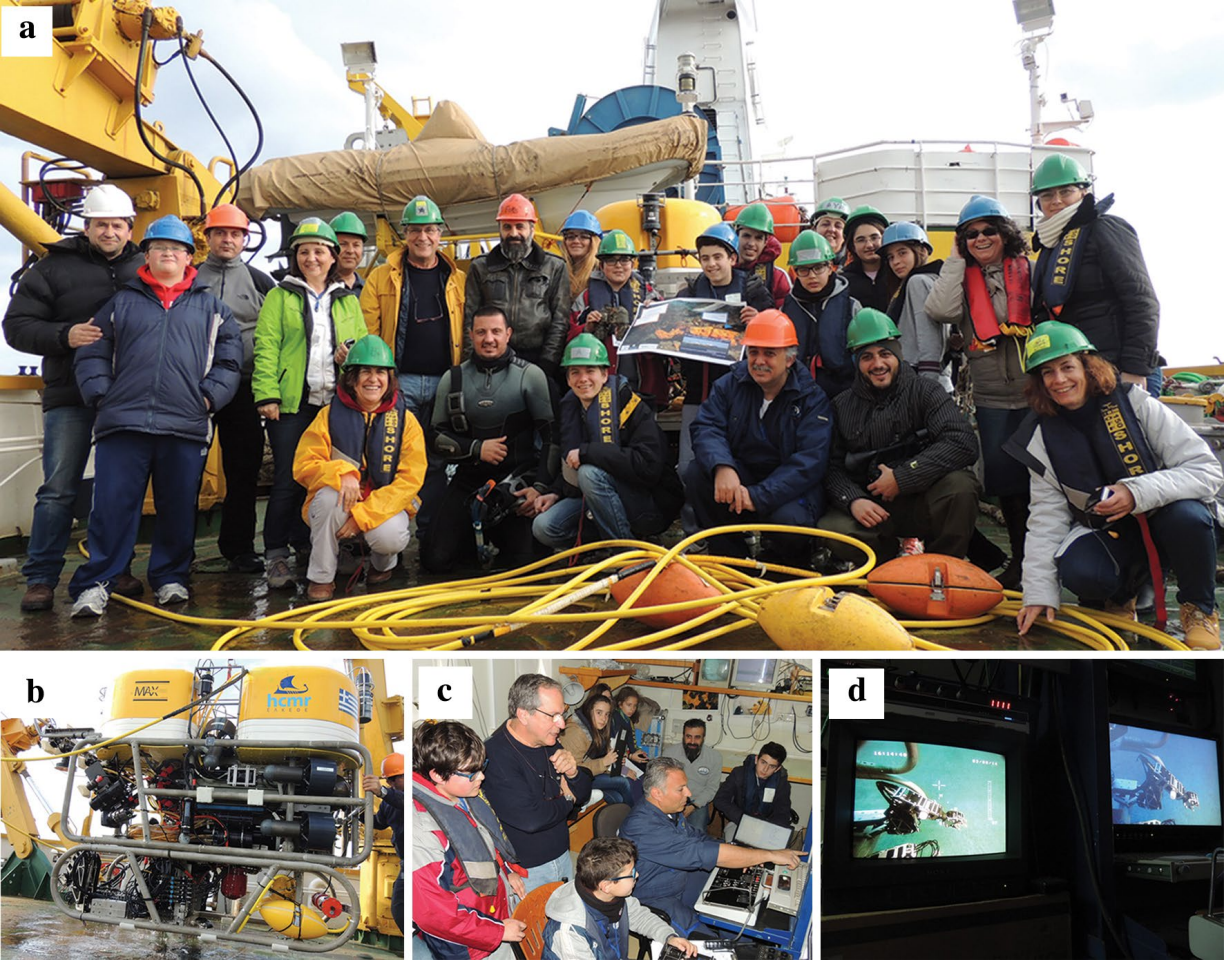
**a** The *Andromeda I* team at the rear deck of the R/V Aegaeo; **b** HCMR’s Max Rover MARK II ROV used in the educational research mission; **c** HCMR’s experienced researchers together with the Underwater Operations Team guiding schoolchildren into handling the ROV; **d** the visual census coming from the ROV’s underwater cameras through which marine litter were recorded

The visual census of marine litter was done by schoolchildren, while being onboard HCMR’s R/V Aegaeo. Experienced scientists accompanied the schoolchildren while surveying marine litter (Fig. 1). Two trial surveys (Table 1) were carried out in selected locations in the eastern part of the Saronikos Gulf (Greece), where benthic marine litter could not be assessed by commercial trawl fisheries and/or by scuba diving. The locations, at the eastern end of the Gulf, were selected based on their proximity to touristic coastal areas, adjacent to the metropolitan area of Attica, with intensive marine navigation (commercial and pleasure crafts), in moderate depths ranging 96–113 m; close to the sites where the highest marine litter density (251,250 items/km^2^) was observed by Katsanevakis and Katsarou (2004) and in areas other than the ones surveyed by Ioakeimidis et al. (2014).

**Table 1:** The characteristics of each ROV dive

	Duration (h)	Depth (m)	Length of ROV transect (km)	Average field of view (m)	Total surveyed area (km^2^)
Dive 1	0.48	111–115	0.97	3.0	2.91 × 10^−3^
Dive 2	1.92	94–98	1.39	3.0	4.17 × 10^−3^

A Max Rover MARK II ROV system was used for surveying marine litter. The Max Rover was operated in a live-boat mode, which allowed the ROV and the support R/V to be moved simultaneously. The ROV was deployed by a lifting crane with a release hook, from where it was released in the water. The pilots operated the ROV through the navigation consoles in which a TrackPoint positioning system (Tracklink 10000) and a sonar were included. The ROV has two Ocean ProHD cameras and two Normal video cameras, plus one very good zoom camera, while there is another HD camera and normal video camera for general purposed video. The HD video is recorded in two High Definition recorders.

The whole educational research mission was filmed in cooperation with the Directorate of Educational Television (Ministry of Culture, Education and Religious Affairs) and three 10-min videos were created. The corresponding videos will be further used as educational material i.e. documentaries.

### Study area

**Fig. 2:**
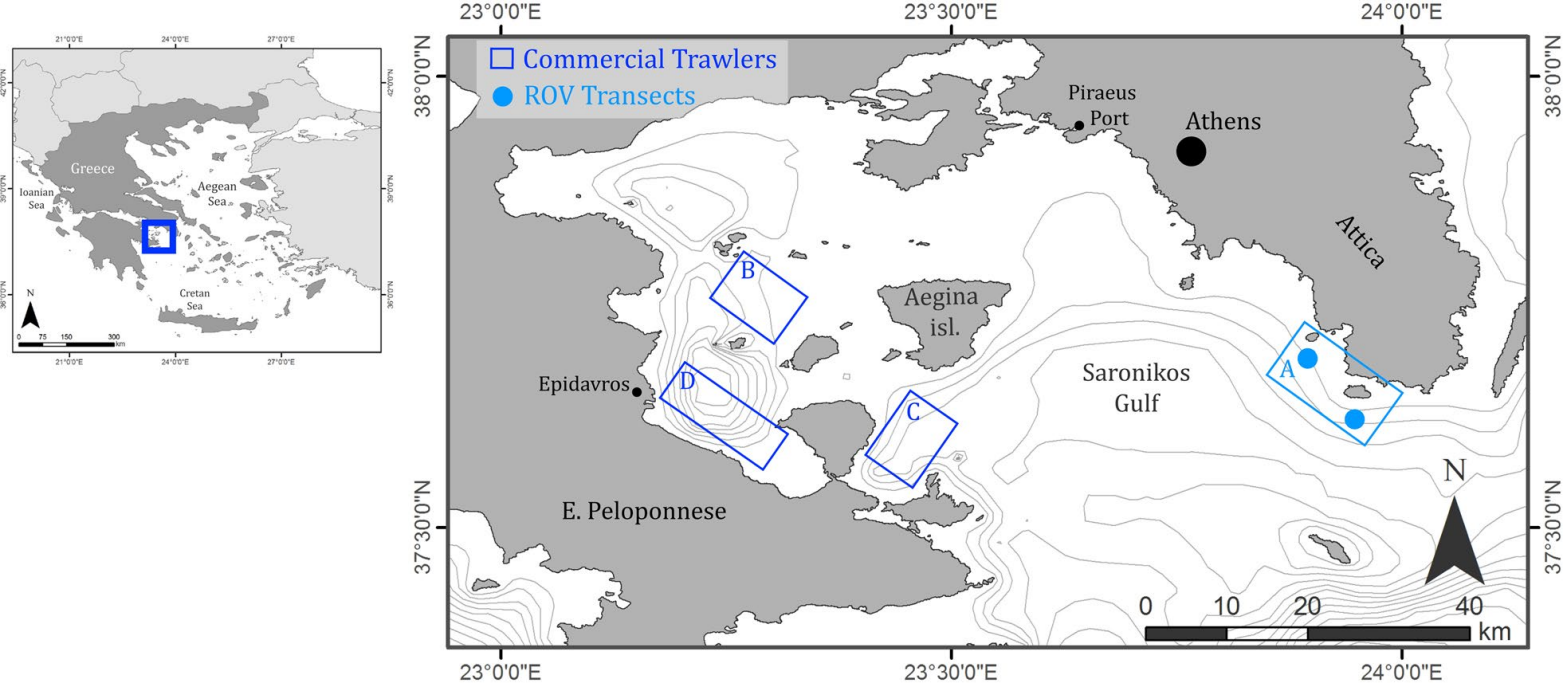
Overview of the marine litter studies in Saronikos Gulf (Greece). (*a*) The area where the assessment of marine litter was done with ROV; (*b*–*d*) the areas where the assessment of marine litter was done by commercial trawl fisheries (Ioakeimidis et al. 2014; 41 hauls)

The Saronikos Gulf (Fig. 2), is located in the South Aegean Sea. It is a semi-enclosed gulf (2600 km^2^), which practically constitutes the sea border of the metropolitan city of Athens and the shore outskirts (Attica region; approx. 4 million inhabitants). The Piraeus city-port (~17,525 arrivals in 2013; www.olp.gr) is located at its northeastern edge. The Saronikos Gulf is considered as one of the most heavily polluted areas of Greece with major solid-waste sources and it is characterized by extreme marine navigation, tourism and well-developed fisheries (commercial and recreational) (Ioakeimidis et al. 2014).

### Data analysis

**Fig. 3:**
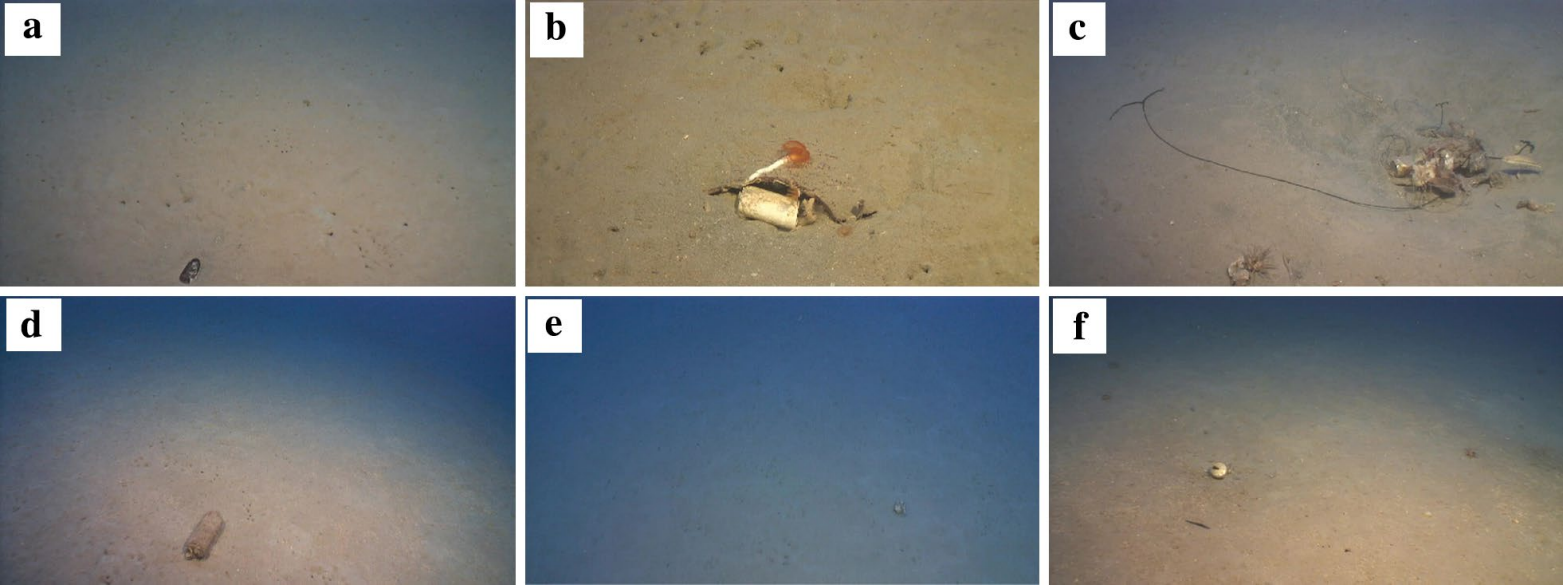
Representative marine litter items that were recorded with the ROV during the first dive: **a** a glass bottle; **b** a beverage metal can covered by a ceramic tile having a polychaeta attached on it; **c** two beverage cans, a fishing longline and two plastic sheets creating a shelter for an echiura (*Bonellia viridis*); **d** a plastic bottle; **e** a metal food can; **f** a beverage metal can

**Fig. 4:**
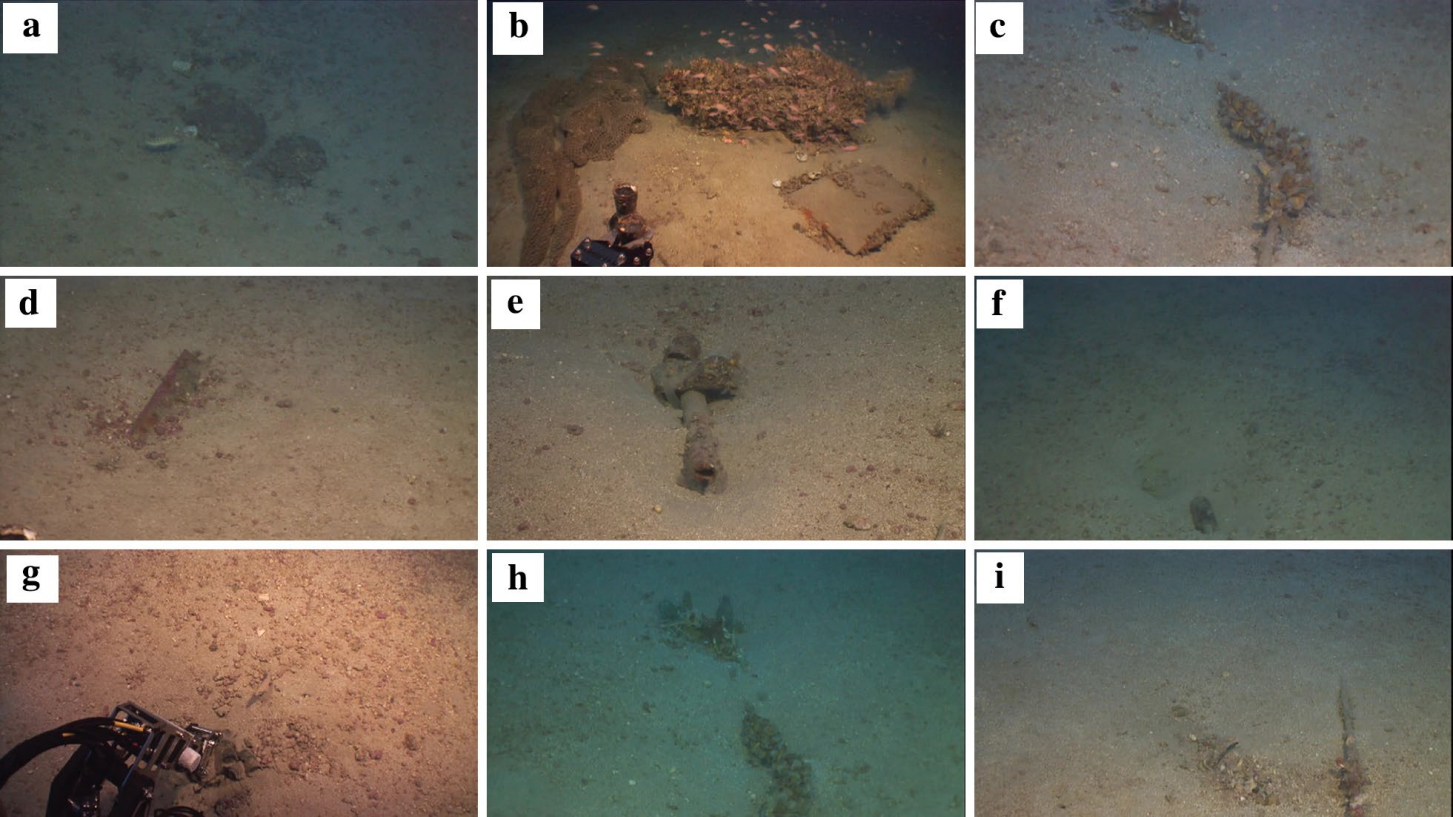
Representative marine litter items that were recorded with the ROV during the second dive: **a** a beverage can and a plastic cup; **b** a derelict bottom-trawl sac and a door lying next to a big rock; **c** a rope embedded in the seafloor sediment with horse mussel (*Modiolus barbatus*) attached on them; **d** a big plastic piece surrounded by maërl beds (*Rhodoliths*), an essential habitat of the circalittoral zone; **e** an iron tube embedded in the sediment and two plastic bottles; **f** a glass bottle; **g** the arm of the ROV is about to hook the so-called “Treasure of Andromeda”, a pair of binoculars embedded in the sediment and surrounded by *Rhodoliths*; **h** a rope with horse mussel (*Modiolus barbatus*) attached on them, a glass bottle, a fishing longline and a plastic sheet; **i** a metal tube embedded in the sediment with polychaeta attached on it

Marine litter item classification was done according to the TGML Master List of categories (Galgani et al. 2013) and a corresponding log-sheet was created dedicated to the ROV dives. Marine litter items were classified according to their material type into the following categories: (1) plastic, (2) metal, (3) rubber, (4) glass/ceramics, (5) natural products, (6) miscellaneous with a total of 32 subcategories. For accuracy reasons, the videos were viewed meticulously, in order to ensure that the marine litter items identification was done precisely (Figs. 3, 4).

A value corresponding to the number of marine litter items per surveyed km was determined (items/km). The covered area was calculated by multiplying the linear distance of the ROV transects on the seafloor by the average field of view of the ROV’s camera i.e. 3 m. Subsequently, the litter density was converted into items/km^2^, in order to enable a comparison with already published results from the Saronikos Gulf (Ioakeimidis et al. 2014; Katsanevakis and Katsarou 2004).

In addition, a classification into different size categories was also completed, empirically, through visual observation into the following six successive size categories: (1) <5 × 5 cm, (2) <10 × 10 cm, (3) <20 × 20 cm, (4) <50 × 50 cm, (5) <100 × 100 cm, (6) >100 × 100 cm. Each category refers to the surface area of each marine litter item. In conclusion, a comparison of the existing marine litter studies for the Saronikos Gulf was attempted with several factors affecting the marine litter abundance. The Pearson correlation coefficient was used in order to link marine litter abundance with (1) distance from the coastline; (2) water depth; (3) distance form population centers.

## Results

**Fig. 5:**
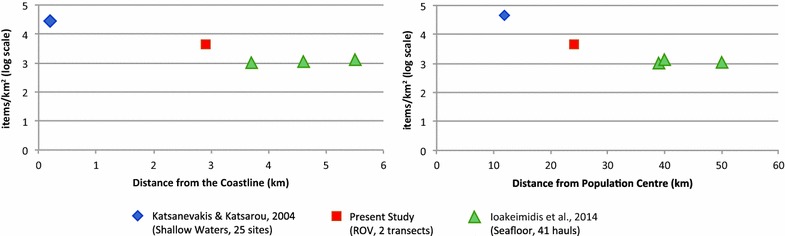
Marine litter density (items/km^2^) in the Saronikos Gulf plotted against: proximity to the coastline (km) and distance from the population nuclei of Athens/Piraeus

In total, 32 marine litter items were found. Twelve marine litter items were recorded during the first dive (0.97 km) and another 20 items during the second dive (1.39 km). The average recording rate was 22.7 items/km (Dive 1: 12.4 items/km; Dive 2: 45.4 items/km) giving a mean marine litter density of 4460 items/km^2^ (Dive 1: 4123 items/km^2^; Dive 2: 4796 items/km^2^). The combination of the scuba diving data from the shallow water along the Saronikos Gulf coastline (23 scuba diving sites; Katsanevakis and Katsarou 2004), the bottom-trawl data from the deeper part of the Gulf (41 hauls; Ioakeimidis et al. 2014) and the two ROV dive observations from the present study suggests that there is a decreasing trend with respect to marine litter abundance to the corresponding distance from the coastline (Fig. 5a). The Pearson correlation coefficient shows high negative correlation between marine litter density and distance from coastline (r = −0.908). In order to distinguish the effects of distance from the coastline and distance from the metropolitan complex of Athens/Piraeus; marine litter density vs. distance from the population centers was also plotted (Fig. 5b). A clear decreasing trend of litter density with distance from the population centers was also observed but with lower correlation (r = −0.824) compared to that of distance from the coastline. The composition of the different marine litter items is given in the following Table 2, for both trial ROV surveys.

**Table 2:** Categories, count and percentage for the 32 marine litter items recorded with the ROV, during the “Andromeda I” educational cruise in the Saronikos Gulf

	Dive 1		Dive 2		Average
	# items	%	# items	%	%
A. Plastics	6	50	12	60	55
B. Metals	5	42	6	30	36
C. Rubber	0	0	0	0	0
D. Glass/ceramics	1	8	1	5	7
E. Natural products	0	0	0	0	0
F. Miscellaneous	0	0	1	5	3

Plastics were the most abundant marine litter type, exceeding 50 % in both surveys. Metals were the second most abundant marine litter type, of significant occurrence. Glass/Ceramics and Miscellaneous only had a small share in the total marine litter collection. Among plastics, bottles (Dive 1: 8.3 %, Dive 2: 15 %), sheets (Dive 1: 25 %, Dive 2: 25 %), bags (Dive 1: 8.3 %), entangled longlines (Dive 1: 8.3 %, Dive 2: 5 %), synthetic rope (Dive 2: 5 %), fishing nets (Dive 2: 5 %) had the highest contribution. Among metals, beverage cans (Dive 1: 41.7 %, Dive 2: 15 %) and fisheries related items (Dive 2: 5 %) were predominant.

**Fig. 6:**
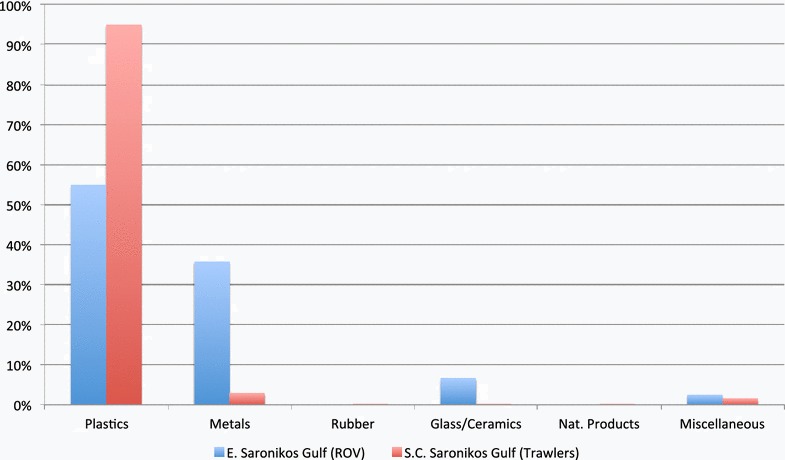
Composition (%) of marine litter items according to their material of use observed by ROV in the Eastern Saronikos Gulf and collected by commercial trawlers in Central and Southern Saronikos Gulf (Ioakeimidis et al. 2014)

In Fig. 6 which is shown below, an initial comparison of the composition of marine litter items is given between the assessment with the ROV in the Eastern Saronikos Gulf and the collected litter by commercial trawl fisheries in the Central and Southern Saronikos Gulf (Ioakeimidis et al. 2014).

Another parameter that was investigated was the variation of size distribution among the different marine litter items. As marine litter items were observed through the video, the sizing classification was done with approximation. The majority (80.0 %) of the marine litter items were classified as medium sized items (>5 × 5 cm up to <50 × 50 cm). Considerable amounts (D1: 6.7 %, D2: 5 %) of small sized items (<5 × 5 cm) were also found, probably due to fragmentation.

With respect to sources; marine litter items were categorized into fisheries and non-fisheries related items. The most common fisheries related marine litter items that are found on the sea floor are: monofilament and entangled fishing lines, synthetic ropes, fishing nets, fishing gear, boots, bobbins and tires re-used as fishing boat fenders. In the first dive, 8.3 % were linked to fisheries while the rest 91.7 % was non-fisheries related. Respectively, in the second dive, 16.7 % was linked to fisheries and the rest 83.3 % was non-fisheries related.

Megafauna was associated with various marine litter items. Several organisms attached on marine litter were recorded i.e. Polychaeta (*Sedentary tubiculous*, *Calcarius worms*, *Serpulidae*), Echiura (*Bonellia viridis*), Gastropoda, Ascidia (*Ascidians*), Bivalvia (*bivalves*), Serpulidae, Anthozoa (*Alcyonium*), Rhodophyceae. Moreover the benthic environment surrounding the marine litter items was very interesting with various recorded organisms i.e. Gastropoda, Echinodermata (*Echinoidea*), Rhodophyceae, Anthozoa (*Anemonia*), Echinoedea (*Anthozoa*), Asteroidea (*Marthasterias*). The substrate of the seafloor in most images (Fig. 4a, d, e, g) corresponds to muddy-sandy sediments and where calcareous red algae (Rhodophyceae) are identified as epiphytic forms on encrusted stones the habitat could be identified as maërl beds (Rhodoliths), an essential habitat of the circalittoral zone. Other biogenic detritus, shells and tube fragments, were also recorded. Approximately 75 % of the marine litter items were associated with epibenthic megafauna. It should be noted that one of the most important impact of litter on marine biota is the alteration of the texture of the seabed (artificial hardgrounds) and thus the change of the structure of benthic communities (Katsanevakis et al. 2007). This impact could be a considerable threat in the future.

Schoolchildren enjoyed the whole mission and showed exceptional maturity and attention during the 2-days research cruise and especially while being in the ROV control room. They identified and classified marine litter properly, according to their material. While inspecting the seafloor and finding marine litter at depths of approx. 100 m they all wondered how do litter exist in areas that are not adjacent to the coastline. This was the hint in order to start explaining to the schoolchildren how does marine litter travel into the seas, offshore in long distance form the coastline or even change waters. Special attention was paid on the sources and all of them realized that the possible solution to the problem is there; the sources. They realized, in the best possible way, that “Just because you can’t see it, it doesn’t mean it isn’t there”. From the reports prepared by the schoolchildren, which participated in the “Andromeda I” educational research cruise, we realized the true care of the children about all the environmental issues concerning the littering of the seas.

## Discussion

The assessment of marine litter was carried out in selected locations on the seafloor of the Saronikos Gulf (Greece) with the use of ROV within the framework of the “Andromeda I” educational research cruise in order to enhance the environmental awareness of schoolchildren. Later, the derived data were used in order to supplement the existing benthic litter data collected by both commercial trawl fisheries (50–350 m) (Ioakeimidis et al. 2014) and scuba diving (<25 m) (Katsanevakis and Katsarou 2004).

Along with the assessment of marine litter, a hands-on experience was offered to schoolchildren that participated in the educational research cruise. The children have a key role since they represent the future citizens who will develop behaviors, and make decisions and shape the future of our seas and oceans. Thus, enhancing children’s environmental awareness is considered crucial for future positive environmental changes to come (Hartley et al. 2015). Within the framework of “Andromeda I”, schoolchildren realized, more clearly than ever, that marine litter is a major environmental problem in which human behavior plays a major role. The whole project decisively contributed in strengthening their awareness on marine litter as well as altering their whole perception on littering and waste. More specifically, they realized marine litter’s negative impact on the marine environment; they clearly defined the causes and understood the importance and longevity of plastic in marine littering. Their concern and interest was recorded through their oral and written evaluation and was confirmed after completing the mission by taking initiatives in order to inform their school community in respect to the protection of the marine environment. In this context, the videos collected from and during Andromeda I educational research cruise, are considered to be a novel best practice and a unique tool to confirm the concern of the schoolchildren for marine litter environmental issue.

Although the number of the collected samples and the survey area itself is limited and thus might make the derived results vulnerable to local variation, an effort was done towards assessing marine litter abundance based on the already elaborative study done for the Saronikos Gulf by Ioakeimidis et al. (2014). A common unit (items/km^2^) is considered of major importance in order to have homogeneous and comparable results and a possible compensation for this problem is introduced. The average litter density (4460 items/km^2^) obtained with ROV was higher than the ones obtained by the commercial trawl fisheries (1214 items/km^2^) but still comparable with the corresponding maximum marine litter density (3428 items/km^2^) (Ioakeimidis et al. 2014). Nonetheless, the litter density from the ROV was almost eightfold lower than the ones recorded in shallow water (31730 items/km^2^) (Katsanevakis and Katsarou 2004). The higher marine litter density reported by the ROV surveys compared to those collected at the southern part of Saronikos Gulf at the same water depths (Area B: 70–120 m) was not surprising as the ROV dives took place in areas adjacent to the coast of Athens/Piraeus, where population nuclei and intense marine navigation are present.

However, the comparison of benthic marine litter in different areas collected with different means is a very challenging task with various uncertainties arising mainly related to the local variations in marine litter abundance and distribution. Nonetheless, such a comparison will give us a better perspective of marine litter abundance in all the compartments of the Saronikos Gulf. The lower densities observed in marine litter derived from the commercial trawl fisheries, to the ones derived from ROV can be also attributed to the great variability on the benthic marine litter spatial distribution as well as to possible litter underestimation arising when comparing the commercial trawlers with the direct ROV litter observations, particularly for the small-sized items. On the other hand, ROVs have also been criticized for possible underestimation of marine litter since buried items are not quantified as well as the limited visibility of some ROV images can seriously affect our ability to detect litter items.

Spengler and Costa (2008) mentioned that due to mesh size and the opening of the cod-end, marine litter may be lost during the return of the net, back to the vessel and therefore the litter density can be underestimated. Moreover, the litter spatial distribution within the fishing grounds can be heavily modified by bottom trawling and a large amount of litter can be removed from the seafloor due to intense trawling. The use of ROVs in selected locations, where commercial trawlers were used for the assessment of benthic marine litter should be highly promoted in order to clarify that reflection. Nonetheless, ROVs cannot be applied for big transects, thus the careful plan of the experimental design is considered of major importance, in which randomness is an essential feature.

A synthesis of the three litter data sets obtained by the three different approaches (Scuba, ROV and trawlers), targeted at different areas of the Saronikos Gulf showed a significant decreasing trend of benthic marine litter density with distance from coastline and distance from the population centers. The lower correlation between litter density and distance from important population nuclei compared to that between litter density and distance from coastline is probably due to the lack of shallow water litter data just off the shore of the Athens/Piraeus population nuclei. The proximity of a highly populated metropolitan area at the north of the Gulf seems to play a dominant role in littering. This is in accordance with the high contribution of non-fisheries related litter (83.3–91.7 %) observed by the ROV.

The “Andromeda I” educational research cruise, implemented within the PERSEUS (FP7) Research Project, proves that marine research and environmental education can be successfully combined towards a common target: Healthy Oceans.

## Conclusions

- Marine litter lying on the seafloor considerably varies in respect to its spatial distribution. This is the case of the Saronikos Gulf (Greece), where plastics are predominant.
- The use of different assessment methods for a same study area with different characteristics (i.e. commonly used fishing grounds vs areas of intense navigation), gives a better view of the marine litter load and distribution. This is also reflected on the corresponding sourcing of the marine litter items.
- ROVs’ cannot only be used for the assessment of marine litter in deep water, but also for assessing marine litter in shallow waters, where commonly used survey techniques (commercial and experimental trawlers) are excluded.
- Schoolchildren are highly worried on marine litter, they are aware of most of their effects on marine environment and biota and they might act as pressure lever towards the alteration of wasting habits for the future generations.
